# Severe Hypokalemia Secondary to Transient Distal Renal Tubular Acidosis in a Previously Healthy Woman

**DOI:** 10.7759/cureus.12765

**Published:** 2021-01-18

**Authors:** Efthymia Kallistrou, Nalini N Architha, Soubhik K Pal, Samson O Oyibo

**Affiliations:** 1 General Medicine, Peterborough City Hospital, Peterborough, GBR; 2 Emergency Medicine, Peterborough City Hospital, Peterborough, GBR; 3 Nephrology, Peterborough City Hospital, Peterborough, GBR; 4 Diabetes and Endocrinology, Peterborough City Hospital, Peterborough, GBR

**Keywords:** hypokalemia, transient, distal renal tubular acidosis, non-gap, hyperchloremia, hypobicarbonatemia, metabolic acidosis, urinary potassium, urinary ph, muscle weakness

## Abstract

Normal anion gap (non-gap) hyperchloremic acidosis with hypokalemia is a medical emergency. There are several causes of this metabolic phenomenon, of which distal renal tubular acidosis is among the very rare causes. In this report, we present an unusual case of a previously healthy woman who was admitted to the intensive care unit with a short history of severe muscle weakness. She had no significant past medical history and was not taking any regular medication. There was also no history of recent drug or herb ingestion. Investigations demonstrated a combination of severe hypokalemia, hyperchloremia, hypobicarbonatemia (non-gap metabolic acidosis), and relatively raised urinary potassium and urinary pH in the presence of severe hypokalemia and metabolic acidosis. Results suggested a diagnosis of distal renal tubular acidosis. The patient responded rapidly to a short course of electrolyte replacement therapy and the condition resolved spontaneously thereafter. This case highlights the fact that distal renal tubular acidosis can occur as a transient phenomenon in previously healthy individuals.

## Introduction

Distal renal tubular acidosis (dRTA) is a rare condition characterized by a failure to acidify the urine in the distal parts of the nephron, including the connecting tubule and the collecting duct. It is due to a transport defect involved in the secretion of hydrogen ions. The defect results in marked acid-base abnormalities, which include hyperchloremic metabolic acidosis and severe hypokalemia, which can be fatal [1,2]. This condition rarely occurs in adults due to infection, drugs, and autoimmune disorders [3]. It is commoner in children where it is usually due to a genetic defect in the acid-base regulatory system of the kidneys [4]. Cases of transient dRTA have been rarely reported in neonates in relation to other metabolic disorders but not in adults. We describe a case of a woman who presented to the emergency department with severe weakness due to severe hypokalemia, which was secondary to transient distal renal tubular acidosis.

## Case presentation

Medical history and demographics

A 66-year-old female presented to the emergency department with an inability to walk. She had noticed muscle ache and progressive weakness in both upper and lower limbs over the preceding two days. There was no associated history of vomiting, diarrhea, fever, seizures, paresthesia, or headache. No cough, breathlessness, or chest pain. There was no history of recent ingestion of any medication, herbal remedies, or recreational substances. Her past medical history included eczema and she was not on any regular medication. She was non-English speaking and had been living in the United Kingdom for the past nine years. She lived with her daughter at the time of the presentation. She reported smoking four cigarettes daily and denied alcohol intake. Clinical examination revealed a healthy-looking female, conscious and alert, with normal body temperature. She had bilateral upper limb weakness (power of 2/5) and lower limb weakness (power of 3/5). There was no sensory deficit. Upper and lower limb muscle tone and reflexes, including plantar reflexes were normal. Other systems, including heart rate and respiratory rate, were normal. Blood pressure and oxygen saturation levels were also normal.

Investigations

**Figure 1:**
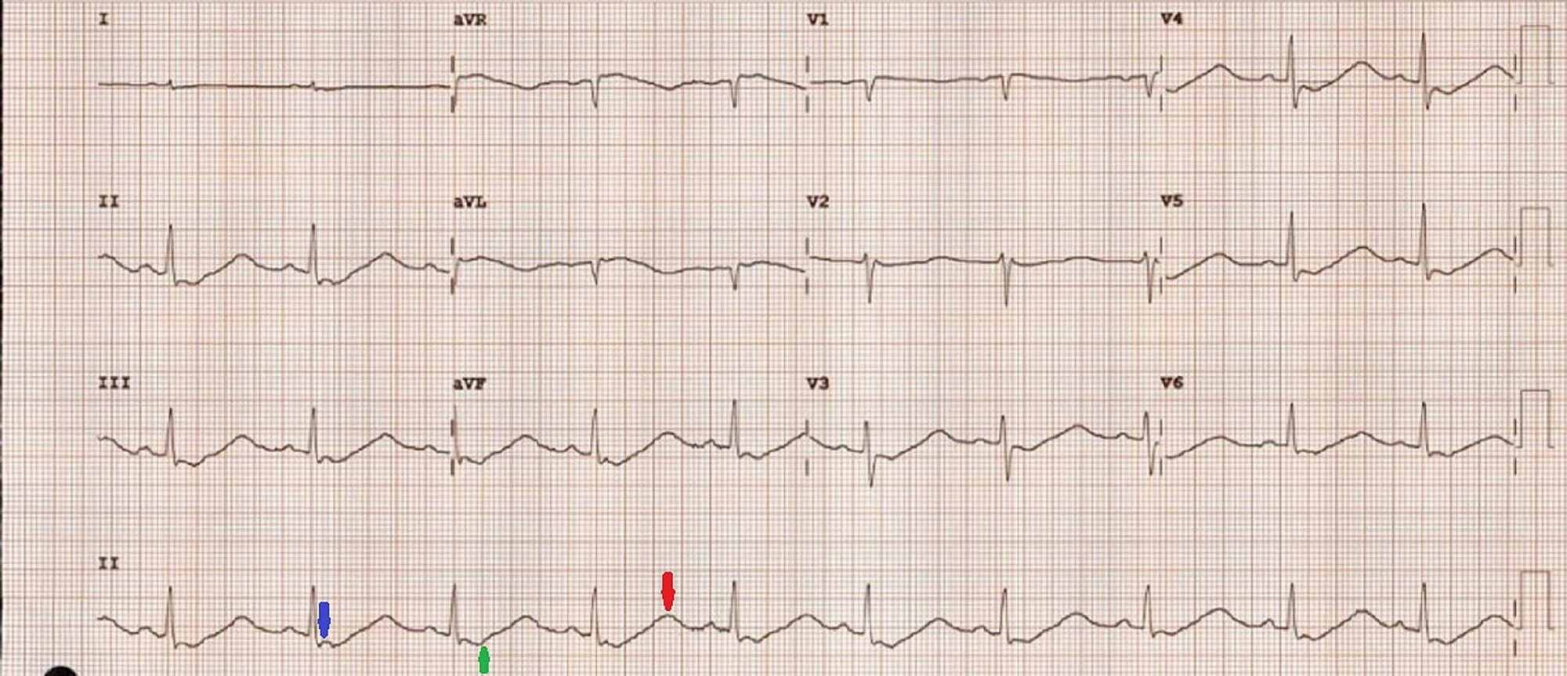
Electrocardiogram performed during admission The initial electrocardiogram showing mild ST depression (blue arrow), inverted T-waves (green arrow), Q-T prolongation and prominent U-waves (red arrow) caused by severe hypokalemia.

The initial investigations revealed severe life-threatening hypokalemia, hyperchloremia, a low serum bicarbonate level, and a low blood pH. A normal full blood count and C-reactive protein indicated the absence of any significant infection. A spot urine analysis showed a urinary potassium level higher than expected (>15 mmol/L) for the severe hypokalemia indicating excessive renal loss of potassium. The urinary pH was also higher than expected (>5.5) for the metabolic acidosis indicating failure to acidify the urine at the same time. Unfortunately, urine chloride and creatinine were not measured so we could not calculate the urinary anion gap or urinary potassium-to-creatinine ratio (Table 1 and Table 2). The patient’s electrocardiogram demonstrated inverted T-waves, Q-T prolongation, prominent U-waves, and mild ST depression secondary to severe hypokalemia (Figure 1). An ultrasound scan demonstrated normal kidneys.

**Table 1:** Laboratory tests, including serum chemistry and venous blood gas analysis

Blood chemistry	Result	Reference range
Sodium (mmol/L)	146	132-145
Potassium (mmol/L)	1.7	3.4-5.1
Chloride (mmol/L)	120	97-110
Bicarbonate (mmol/L)	13	23-30
Anion gap	13	4-16
Creatinine (mmol/L)	82	45-84
Urea (mmol/L)	5.8	2.5-7.8
Serum corrected calcium (mmol/L)	2.4	2.20-2.60
Phosphate (mmol/L)	0.83	0.8-1.5
Magnesium (mmol/L)	1.03	0.7-1.0
Glucose (mmol/L)	5.6	4.0-7.0
Thyroid stimulating hormone (mU/L)	2.3	0.3-4.2
9-am cortisol (nmol/L)	303	250-600
Renin (mU)	4.0	5.4-30
Aldosterone (pmol/L)	<70	90-405
Total protein (g/L)	72	60-80
Albumin (g/L)	45	35-50
Globulin (g/L)	27	20-35
Venous pH	7.305	7.35-7.45
Venous PCO_2_ (kPa)	4.59	4.27-6.40
Venous PO_2_ (kPa)	3.10	4.27-6.40
Venous bicarbonate (mmol/L)	16.7	23-30
Venous lactate (mmol/L)	1.33	0.2-1.8

**Table 2:** Urine spot test

Spot urine test	Result	Expected range
Sodium (mmol/L)	85	
Potassium (mmol/L)	17	<15.0
Urea (mmol/L)	115	
pH	6.0	<5.5
Specific gravity	1.010	1.002-1.020

Urine dipstick test for glucose, ketones, protein, and blood was negative. The coronavirus (COVID-19) screen was negative. The thyroid function, liver function, and overall renal function were normal. Renin-aldosterone levels ruled out Conn’s syndrome (hyperaldosteronism) as a cause of hypokalemia. The combination of hypokalemia, hyperchloremia, hypobicarbonatemia (non-gap hyperchloremic metabolic acidosis), and relatively raised urinary potassium and urinary pH in the presence of severe hypokalemia and metabolic acidosis, respectively, all suggested distal renal tubular acidosis.

Treatment

The patient was treated with intravenous potassium replacement and because of persistent hypokalemia and metabolic acidosis, the patient was transferred to the intensive care unit for replacement via a central venous line and closer monitoring. Her potassium levels normalized after 72 hours of treatment. Over three days of intensive therapy, she required a total of 516 mmol of intravenous potassium and 72 mmol of oral bicarbonate (as sodium bicarbonate). She was discharged shortly afterward with a short course of oral potassium (48 mmol/day) and sodium bicarbonate (24 mmol/day) to last for seven days.

Outcome and follow-up

The patient made a rapid and full recovery and was due to have a repeat serum potassium check a week after stopping the potassium and bicarbonate supplements. Further investigation results revealed normal serum immunoglobulin levels and normal autoimmune antibody screen ruling out autoimmune, auto-inflammatory, and mixed connective tissue disorders (Table 3). The patient’s serum potassium levels remained normal a week, a month, and three months after discharge and the patient remains well.

**Table 3:** Protein electrophoresis and autoimmune workup

Test	Result	Reference range
Immunoglobulin G (g/L)	9.6	6.0-16.0
Immunoglobulin A (g/L)	4.27	0.8-4.0
Immunoglobulin M (g/L)	0.39	0.5-2.0
Anti-nuclear antibody (CU)	4.3	<20.0
Rheumatoid factor (mmol/L)	<12	0.7-1.0
Complement factor 3 (g/L)	0.87	0.75-1.65
Complement factor 4 (g/L)	0.19	0.14-0.54
Other autoimmune antibodies including: Anti-double stranded DNA (anti-dsDNA), Anti-neutrophil cytoplasmic antibody (ANCA), Anti-Sjögren’s syndrome A (anti-SSA) (Ro60), Anti-Sjögren’s syndrome B (anti-SSB) (La), Anti-Jo-1, Anti-ribonucleic protein (anti-RNP), Anti-Smith (Anti-Sm), Anti-scleroderma antibody (anti-Scl-70), Anti-ribosomal-P, Anti-centromere, Anti-Mi-2, Anti-Th/To, Anti-Ku, Anti-RNA polymerase III, Anti-PM/Scl, Anti-proliferating cell nuclear antigen (anti-PCNA), Anti-liver (smooth muscle, mitochondrial) antibodies, Anti-liver kidney microsomal type 1 antibodies (anti-LKM), Anti-glomerular basement membrane antibody (anti-GBM)	Negative	
Protein electrophoresis	Normal, no monoclonal band seen	

## Discussion

Distal renal tubular acidosis (dRTA) is a condition that occurs as a result of impaired urinary acid excretion and inability to lower urinary pH despite the presence of acidosis. This is due to a defect in hydrogen ion secretion by the α-intercalated cells in the distal convoluted tubules and the collecting tubules. Reduced excretion of ammonia ions in the urine is also present in affected individuals. Other significant sequelae of this condition include striking hypokalemia, hyperchloremic non-anion gap (non-gap) metabolic acidosis, hypocitraturia, hypercalciuria, and nephrocalcinosis [1-5]. Muscle weakness and muscle paralysis due to hypokalemia from renal potassium wastage is a prominent symptom [5]. Patients can also experience headaches, lack of energy, nausea, and vomiting as a result of acidosis. Depending on the severity of acidosis, stupor, coma, myocardial instability, or a cardiac arrest may occur. The body attempts to reduce carbon dioxide levels by a compensatory increase in respiratory rate however, in long-standing disease, this may lead to muscle fatigue and respiratory failure. In children, stunted growth and bone loss (rickets, osteomalacia) are often present because of chronic metabolic acidosis. The associated nephrocalcinosis, nephrolithiasis, and hypercalciuria can lead to the development of chronic kidney disease. Hearing loss is seen in distinct autosomal recessive types [5].

Distal renal tubular acidosis can be secondary due to systemic diseases or drug-induced or can be primary due to gene mutations. Several systemic or autoimmune have been associated with acquired dRTA. The underlying mechanism is believed to be related to direct renal tubular damage [5]. Table 4 shows a list of conditions associated with acquired (secondary) dRTA [1-5].

**Table 4:** Conditions associated with acquired distal renal tubular acidosis

Conditions associated with acquired distal renal tubular acidosis	
Autoimmune conditions	Systemic lupus erythematosus (SLE), Sjögren’s syndrome, rheumatoid arthritis, systemic sclerosis, thyroiditis, hepatitis, primary biliary cirrhosis
Hypergammaglobulinemic states	Monoclonal gammopathy, multiple myeloma, amyloidosis, cryoglobulinemia, chronic liver disease
Tubulointerstitial diseases	Chronic pyelonephritis, chronic interstitial nephritis, obstructive uropathy, renal transplant rejection
Genetic conditions	Marfan’s syndrome, Ehler-Danlos syndrome, sickle cell disease, congenital urinary tract obstruction
Drugs	Amphotericin B, lithium, non-steroidal anti-inflammatory drugs, toluene and pentamidine
Miscellaneous	Familial hypercalciuria, chronic hypercalcemia, Wilson’s disease

Primary dRTA can occur sporadically or be inherited in an autosomal dominant or recessive pattern. So far, mutations have been identified in the following genes: the solute carrier family 4 member 1 (*SLC4A1*) gene that encodes a protein called anion exchanger 1 or AE1 that helps negatively-charged atoms cross cell membranes, the ATPase H+ transporting V1 submit B1 (*ATP6V1B1*) gene and the ATPase H+ transporting V0 submit A4 (*ATP6V0A4*) gene that encodes proteins that are part of a proton pump complex called vacuolar ATPase (V-ATPase) that helps positively-charged atoms cross cell membranes, the tryptophan-aspartate repeat domain 72 (*WDR72*) gene that encodes a protein possibly associated with intracellular endocytic vesicle trafficking, and the forkhead box i1 transcription factor (*FOXI1*) gene that encodes a protein involved in regulating the V-ATPase proton pump subunits in the inner ear, kidney, and epididymis [6,7].

The diagnosis of dRTA is based on a demonstration of the nephron’s inability to lower urinary pH below 5.5 in the presence of systemic acidosis, normal anion gap (non-gap), hyperchloremia, and hypokalemia. Measurement of the urinary electrolytes should reveal a positive anion gap that reflects the decrease in urine ammonium excretion and can help differentiate between other causes of metabolic acidosis like diarrhea [5]. An electrocardiogram (ECG) should be performed in cases of moderate or severe hypokalemia to look for changes that can result in life-threatening dysrhythmias. Diagnostic work-up should always include the search for nephrocalcinosis by ultrasonography and the measurement of 24-hour urinary excretion of calcium, magnesium, and citrate. Furthermore, failure to lower urine pH below 5.3, either after ammonium chloride loading or after furosemide administration can establish the diagnosis of dRTA [1,3,8]. If no underlying cause is found then genetic testing will be required.

The treatment for dRTA in the acute setting consists of adequate potassium replacement and correction of the metabolic acidosis. Treatment of the underlying condition cannot be overemphasized. For chronic dRTA, oral bicarbonate replacement as sodium bicarbonate (1-2 mmol/kg/day) or potassium citrate (1-2 mmol/kg/day) is recommended until the plasma bicarbonate increases to at least 22 mmol/L. Citrate salts also correct the hypocitraturia and prevent nephrolithiasis [7,9].

There are other causes of non-gap hyperchloremic metabolic acidosis with hypokalemia [10]. Non-gap metabolic acidosis can also occur with raised or normal serum potassium levels [10]. These causes are summarized in Table 5 below.

**Table 5:** Causes of normal anion gap (non-gap) metabolic acidosis

Causes of non-gap metabolic acidosis		
Conditions with hypokalemia	Gastrointestinal	Diarrhea, intestinal fistulae, laxative abuse, uretero-ileostomy, and uretero-sigmoidostomy
	Metabolic	Ketoacidosis, lactic acidosis, and renal tubular acidosis
	Drugs	Administration of chloride-rich solutions and the use of acetazolamide in patients with renal dysfunction
Conditions with raised or normal serum potassium levels	Administration of hydrogen and chloride ions in the form of total parenteral nutrition or ammonium chloride administration, hyperkalemic dRTA due to hyporeninemic hypoaldosteronism, tubular resistance to aldosterone, aldosterone deficiency, chronic renal failure, Gordon’s syndrome (pseudohypoaldosteronism), and administration of chloride-rich solutions	

There are very few published case reports of transient dRTA. One such case described an elderly female who had transient dRTA after organophosphate poisoning. She had multiple ventricular arrhythmias due to hypokalemia but all resolved after treatment [11]. Another interesting report described a neonate with life-threatening metabolic acidosis due to distal renal tubular acidosis and secondary hyperparathyroidism. This patient was diagnosed with Lightwood-Albright syndrome (transient infantile renal tubular acidosis) [12]. There is also a case report of a young child who had transient hyperkalemic dRTA with bicarbonate wasting with no underlying cause found [13].

We have presented a rare case of severe hypokalemia secondary to transient distal RTA in a previously healthy woman. There was no underlying cause identified for this patient’s presentation, the patient responded rapidly to a short course of electrolyte replacement therapy and the condition resolved spontaneously after a few days.

## Conclusions

Severe life-threatening hypokalemia and hyperchloremic acidosis due to distal RTA can occur as a transient phenomenon. There are other more common causes of non-gap hyperchloremic acidosis that can easily be excluded by adhering to a systematic approach. It is possible that our patient had an underlying cause for transient dRTA; however, despite extensive investigation, no cause was identified. We hope that this case report will not only add to the existing literature but also contribute to the heightened awareness of transient distal renal tubular acidosis.
